# Zahn- und Kieferfehlstellungen – gesundheitliche Relevanz und Behandlung

**DOI:** 10.1007/s00103-021-03372-3

**Published:** 2021-07-08

**Authors:** Sabine Ruf, Peter Proff, Jörg Lisson

**Affiliations:** 1 Poliklinik für Kieferorthopädie, Justus-Liebig-Universität Gießen, Schlangenzahl 14, 35392 Gießen, Deutschland; 2 Poliklinik für Kieferorthopädie, Universitätsklinikum Regensburg, Regensburg, Deutschland; 3 Klinik für Kieferorthopädie, Universität des Saarlandes, Homburg/Saar, Deutschland

**Keywords:** Zahnfehlstellung, Kieferorthopädie, Mundgesundheit, Allgemeingesundheit, Lebensqualität, Malocclusion, Orthodontics, Oral health, General health, Quality of life

## Abstract

Zahn- und Kieferfehlstellungen gehören zu den häufigsten Mundgesundheitsbeeinträchtigungen beim Menschen. Der vorliegende Beitrag gibt eine Übersicht zu deren Ursachen, Häufigkeit und Folgen. Er zeigt die präventiven und kurativen Möglichkeiten kieferorthopädischer Behandlungen auf und gibt Informationen zu deren rechtlichen Rahmenbedingungen in Deutschland. Inanspruchnahme und Qualität der kieferorthopädischen Versorgung werden im internationalen Vergleich dargestellt.

Bei den Ursachen für Zahn- und Kieferfehlstellungen spielen genetische, epigenetische, funktionelle und umweltbedingte Faktoren eine Rolle, die individuell meist nicht eindeutig feststellbar sind. Bisher zeigen nur kleinere Querschnittsstudien, dass bis zu 80 % der Kinder in Deutschland betroffen sind. Essen, Trinken, Kauen, Sprechen und Atmen können beeinträchtigt sein, die Neigung zu Parodontalerkrankungen sowie Überlastungsschäden von Kiefergelenk und Kaumuskulatur sind erhöht. Bei einer Proklination der oberen Schneidezähne steigt die Gefahr von Frontzahntraumata. Fehlstellungen können zudem negative psychosoziale Folgen oder Einschränkungen der Lebensqualität zur Folge haben. Kieferorthopädische Behandlungen leisten in Kooperation mit anderen (zahn-)medizinischen Fachdisziplinen einen wichtigen präventiven bzw. kurativen Beitrag zur Verbesserung der Mundgesundheit, der Allgemeingesundheit und der Lebensqualität.

Die Kieferorthopädie bietet ein erhebliches Potenzial für die Stärkung der zahnärztlichen Prävention im Gesundheitswesen, zumal die gesetzliche Krankenversicherung (GKV) eine breitflächige Versorgung der Bevölkerung mit kieferorthopädischen Leistungen auf international anerkanntem, hohem Niveau ermöglicht. Um die Prävention weiter zu verbessern, wird die Einführung eines kieferorthopädischen Screenings im 7.–8. Lebensjahr als systematische Vorsorge empfohlen.

## Abstract (en)

Malocclusions are among the most common human diseases impairing oral health. The present paper gives an overview on their etiology, prevalence, and consequences. It further presents the corrective and preventive potential of orthodontic treatment and gives information on the treatment-associated legal framework in Germany. The use and quality of the orthodontic health service will be described in the international context.

The etiology of malocclusions has genetic, epigenetic, functional, and environmental components, which can seldomly be differentiated on the individual level. Previous small, cross-sectional studies show that up to 80% of children in Germany are affected. Eating, drinking, chewing, speaking, and breathing may be impaired and the predisposition for periodontal disease and overuse injuries of the temporomandibular joint and the masticatory musculature increase. A proclination of the upper incisors increases the risk for dental trauma. Furthermore, malocclusions may have negative psychosocial effects and impair quality of life. In cooperation with other dental and/or medical disciplines, orthodontics make positive preventive and curative contributions to oral health, general health, and quality of life.

Against this background, orthodontics has a substantial potential for the enhancement of dental prevention in healthcare. This is especially true as the German social health insurance (GKV) allows for a widespread orthodontic treatment coverage of the population at an internationally accepted, high-quality level. To further improve prevention, the implementation of a systematic orthodontic screening at the age of 7–8 years in the form of a systematic preventive measure is recommended.

## Einleitung

Mundgesundheit, Allgemeingesundheit und Lebensqualität sind untrennbar miteinander verbunden [1]. Erkrankungen des Kauorgans, also der Zähne, Kiefer, Kiefergelenke und Kaumuskulatur, können die Allgemeingesundheit direkt beeinträchtigen, indem sie Schmerzen und Leid verursachen, die Nahrungsaufnahme bzw. Nahrungswahl beeinflussen, das Sprechen erschweren und somit das Wohlbefinden und die Lebensqualität reduzieren. Erkrankungen des Kauorgans können sich aber auch indirekt auf die Allgemeingesundheit auswirken – vielfältige Zusammenhänge mit chronischen Erkrankungen sind bekannt (z. B. Diabetes, Herzerkrankungen, Schlaganfall; [2]), getreu dem Motto des Tages der Zahngesundheit 2009: „Gesund beginnt im Mund – krank sein manchmal auch.“

Zahn- und Kieferfehlstellungen gehören neben Karies und Gingivitiden (Zahnfleischentzündungen) zu den häufigsten Mundgesundheitsbeeinträchtigungen des Menschen [3, 4]. Durch eine kieferorthopädische Behandlung können sie korrigiert und mögliche funktionelle Folgen beseitigt oder verhindert werden. Deshalb ist die Betrachtung von Zahn- und Kieferfehlstellungen im Hinblick auf eine synoptische (zahn-)medizinische Versorgung der Bevölkerung als auch für die Prävention essenziell.

Die Behandlung von Zahn- und Kieferfehlstellungen ist Aufgabe des Fachgebietes Kieferorthopädie und umfasst Erkennung, Verhütung und Behandlung von Fehlbildungen des Kauorganes, Zahnstellungs- und Bissanomalien, Kieferfehlbildungen und Deformierung der Kiefer und des Gesichtsschädels im gesamtmedizinischen Kontext. Fachzahnärzte für Kieferorthopädie sind durch eine vierjährige Weiterbildung für dieses Fachgebiet spezialisiert [5].

Im Idealfall passen die Zähne von Ober- und Unterkiefer in allen 3 Raumebenen perfekt zusammen. Abweichungen vom Ideal können einzeln als auch in Kombination von Zahn- und Kieferfehlstellungen vorkommen. Ihr Ausprägungsgrad ist äußerst variabel. Zu den häufigsten Abweichungen gehören Breitendiskrepanzen (z. B. Kreuzbisse) und Längendiskrepanzen (z. B. Distalbisse oder Mesialbisse) sowie Störungen im Durchbruch oder in der Einstellung einzelner bzw. mehrerer Zähne.

Der vorliegende Übersichtsartikel beleuchtet die Ursachen, Häufigkeit und Folgen von Zahn- und Kieferfehlstellungen. Ferner zeigt er die präventiven und kurativen Möglichkeiten kieferorthopädischer Behandlungen auf und gibt Informationen zu den rechtlichen Rahmenbedingungen der kieferorthopädischen Behandlung in Deutschland und deren Inanspruchnahme sowie zur Qualität der kieferorthopädischen Versorgung im internationalen Vergleich.

## Ursachen

Die Ursachen für Zahn- und Kieferfehlstellungen sind multifaktoriell. Dabei spielen genetische, epigenetische, funktionelle und umweltbedingte Faktoren eine Rolle. Auf individueller Ebene ist der ätiologische Hintergrund meist nicht eindeutig feststellbar [6, 7].

Während sich genetische und epigenetische Faktoren durch eine kieferorthopädische Behandlung nicht primär therapeutisch beeinflussen lassen, besteht bei funktionellen Faktoren (z. B. Fehlfunktionen der Zungen‑, Lippen‑, Wangen- und Kaumuskulatur) und umweltbedingten Faktoren (z. B. vorzeitiger Verlust von Milch- oder bleibenden Zähnen, Zahntraumata) die Chance auf eine kausale oder präventive Therapie. Die Prognose für den Behandlungserfolg bei Zahn- und Kieferfehlstellungen sowie die Stabilität der Ergebnisse ist bei überwiegend funktionellen bzw. umweltlichen Ursachen besser als bei einem genetischen Hintergrund. Daher setzt eine präventionsorientierte Kieferorthopädie eine frühzeitige Erkennung aller funktionellen und umweltbedingten Faktoren voraus, die das kraniofaziale Wachstum und die Gebissentwicklung negativ beeinflussen können [8].

## Häufigkeit

Zahn- und Kieferfehlstellungen sind häufig und gehören, wie bereits oben erwähnt, neben Karies und Gingivitiden zu den häufigsten Mundgesundheitsbeeinträchtigungen des Menschen [3]. Ihre Häufigkeit weist globale Schwankungen auf [4]. Aktuelle bevölkerungsrepräsentative Daten für Kinder und Jugendliche aus Deutschland fehlen, denn Zahn- und Kieferfehlstellungen wurden zuletzt im Jahr 1989 im Rahmen der ersten Deutschen Mundgesundheitsstudie (DMS I) erfasst [9]. Gemäß kleinerer Querschnittstudien aus verschiedenen Bundesländern weisen bis zu 80 % der untersuchten Kinder Zahn- und Kieferfehlstellungen auf [10–13]; ein objektiver Behandlungsbedarf besteht in 41–52 % der Fälle [11, 12].

## Folgen von Zahn- und Kieferfehlstellungen

Zahn- und Kieferfehlstellungen können das Essen, Trinken, Kauen, Sprechen und die Atmung direkt beeinträchtigen [14–16], weil sie u. a. die Okklusion und Artikulation der Zähne, also den Kontakt und die Gleitbewegungen zwischen den Zähnen von Ober- und Unterkiefer, z. B. in Form von Zwangsführungen oder durch eine falsche Lage der Kiefer zueinander stören, den Mundschluss beeinträchtigen oder die Lautartikulationszonen verändern. Ferner können sie Überlastungsschäden des Kiefergelenks und/oder der Kaumuskulatur bedingen und somit auch indirekt das Essen, Trinken, Kauen, Sprechen und die Atmung beeinträchtigen [17]. Des Weiteren konnten Studien zeigen, dass die häufig vorkommende Proklination (Vorstehen) der oberen Schneidezähne die Gefahr von Frontzahntraumata erhöht [18] und dass eine Assoziation zwischen Zahn- und Kieferfehlstellungen mit Parodontalerkrankungen besteht [19]. Nicht zuletzt können Zahn- und Kieferfehlstellungen negative psychosoziale Folgen haben, die mundgesundheitsbezogene Lebensqualität verringern [20, 21] oder, bei umfangreicher Ausprägung, zu Mobbing in der Schule führen [22].

Neben der Art bestimmt vor allem das Ausmaß einer Zahn- und Kieferfehlstellung deren Folgen, d. h., je größer das Ausmaß der Abweichung von einem normalen Gebiss ist, desto mehr funktionelle und psychosoziale Auswirkungen der Fehlstellung sind prinzipiell zu erwarten [21, 23, 24]. Allerdings gibt es Patienten, die z. B. aufgrund einer höheren psychischen Widerstandsfähigkeit (Resilienz) besser als andere mit einer Fehlstellung umgehen können. Daher unterliegen die Folgen von Zahn- und Kieferfehlstellungen einer hohen interindividuellen Variabilität und müssen für jeden Patienten individuell beurteilt werden.

## Kieferorthopädie als präventive und kurative Maßnahme

Bis dato ist das Evidenzniveau zum Effekt kieferorthopädischer Behandlungen auf die Mundgesundheit vergleichsweise niedrig [25]. Gründe liegen beispielsweise darin, dass die Mundgesundheit durch multiple Faktoren beeinflusst wird und nicht allein von der Durchführung kieferorthopädischer Maßnahmen abhängt. Außerdem schränken die langen Latenzzeiten (Jahre bis Dekaden) verschiedener Einflussfaktoren der Mundgesundheit sowie die langsame Progression der häufigsten oralen Erkrankungen (Karies, Parodontalerkrankungen, Mundschleimhauterkrankungen) die wissenschaftliche Untersuchung präventiver und therapeutischer kieferorthopädischer Effekte ein. Der wissenschaftliche Nachweis des kausalen Effektes einer kieferorthopädischen Behandlung würde zudem eine randomisierte klinische Studie mit unbehandelter Kontrollgruppe erfordern, was vor dem Hintergrund der langen Latenzzeiten weder ethisch noch finanziell/administrativ möglich ist [26]. Erfreulicherweise wurde die Untersuchung von Zahn- und Kieferfehlstellung in die DMS VI wieder aufgenommen, sodass zukünftig Rückschlüsse auf der Basis bevölkerungsrepräsentativer Querschnittsergebnisse möglich sein werden.

Die Literatur liefert, trotz der oben genannten Schwierigkeiten, eindeutige Hinweise, dass kieferorthopädische Behandlungen auf verschiedenen medizinischen Wirkebenen präventive Effekte haben, welche die Morphologie, die Funktion und die Entwicklungsprozesse des Gebisses und der Kiefer bzw. des Gesichts betreffen. Dazu gehört die Reduzierung des Risikos für Frontzahntraumata, Karies und Gingivitiden sowie die Beseitigung oraler Dysfunktionen und Habits (schädliche Angewohnheiten; [25, 27]).

Auf der kurativen Ebene zeigen kieferorthopädische Behandlungen neben einer Verbesserung der mundgesundheitsbezogenen Lebensqualität [20] beispielsweise therapeutische Effekte hinsichtlich einer Verbesserung der Nasenatmung [27, 28], wodurch unter anderem die Karies- und Gingivitisneigung sowie die Anfälligkeit für infektiöse Atemwegserkrankungen, Asthma und allergische Rhinitiden reduziert wird. Durch kieferorthopädische Behandlungen kann ferner der nasopharyngeale Luftraum, also der Atemweg, vergrößert werden, wodurch Schnarchen und Schlafapnoen reduziert oder sogar beseitig werden können. Auch die Kaueffektivität und Kaueffizienz werden durch die Harmonisierung der Zahnbögen und die Korrektur fehlender bzw. verlagerter Zähne gesteigert [27]. Als positiver Nebeneffekt der besser zerkleinerten Nahrung wird die Magenentleerung in den Darm beschleunigt [29], was sich wiederum positiv auf gastrointestinale Symptome auswirken kann. Des Weiteren trägt die Beseitigung von Zahn- und Kieferfehlstellungen zur erfolgreichen Therapie von Sprechstörungen bei [30].

In Kooperation mit den anderen zahnärztlichen Fachdisziplinen schaffen kieferorthopädische Behandlungen von Zahn- und Kieferfehlstellungen grundlegende oder verbesserte Voraussetzungen für die Einordnung retinierter Zähne, für Zahntransplantationen, prothetische Versorgungen und parodontologische Behandlungen und sorgen somit für einen verlängerten Zahnerhalt, eine verlängerte Lebensdauer prothetischer und konservierender Versorgungen und den langfristigen Erhalt einer optimalen Kaufunktion. Ferner ist die Kieferorthopädie unverzichtbarer Bestandteil interdisziplinärer Therapien in Kooperation mit diversen medizinischen Fachgebieten u. a. bei Patienten mit Lippen-Kiefer-Gaumen- und Segelspalten, kraniofazialen Syndromen, obstruktiven Schlafapnoen, juvenilen rheumatoiden Arthritiden sowie seltenen Erkrankungen mit kraniofazialen Manifestationen [27].

## Rechtliche Rahmenbedingungen der kieferorthopädischen Behandlung in Deutschland

**Figure 1:**
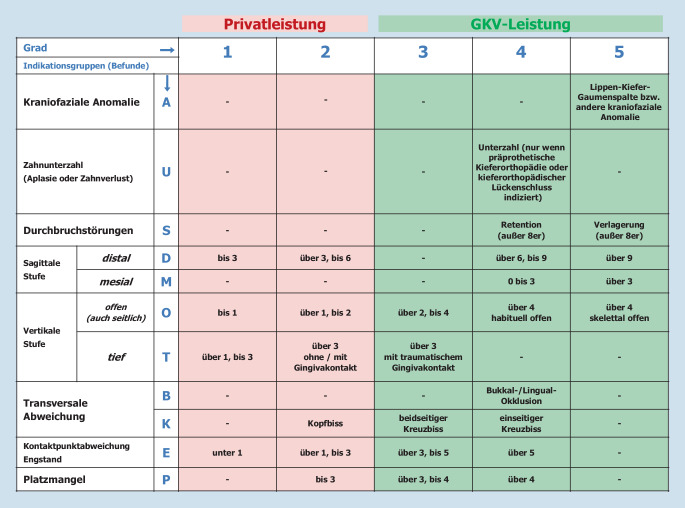

Gemäß Sozialgesetzbuch (§29 Absatz 1 SGB V) haben gesetzlich Krankenversicherte einen Anspruch auf kieferorthopädische Versorgung, wenn medizinisch begründete Indikationsgruppen eines bestimmten Schweregrades vorliegen, bei dem davon auszugehen ist, dass das Kauen, Beißen, Sprechen oder Atmen erheblich beeinträchtigt ist oder beeinträchtigt zu werden droht. Die zur Behandlung zulasten der gesetzlichen Krankenversicherung (GKV) berechtigenden Indikationsgruppen sind alters- bzw. entwicklungsabhängig und tragen somit dem Präventionsfokus im Bereich der Gesundheitsförderung Rechnung. In Abb. 1 sind beispielhaft die Indikationsgruppen für die Hauptbehandlungsphase (Beginn 2. Wechselgebissperiode bis 18. Lebensjahr) wiedergegeben. Vergleichbare überwiegend ausprägungsgradbezogene Indikationsgruppensysteme werden auch in anderen Ländern verwendet. Beispiele sind der IOTN (Index of Orthodontic Treatment Need; [31]), der ICON (Index of Complexity, Outcome and Need; [32]), der SMBI (Swedish Medical Board Index; [33]) oder die schweizerische Liste der Geburtsgebrechen [34].

Jedoch hat das deutsche kieferorthopädische Indikationsgruppensystem (KIG-System) auch Schwächen, welche unter Umständen dazu führen können, dass trotz (zahn-)medizinischer Notwendigkeit eine Behandlung zulasten der GKV nicht möglich ist.

(1) Die Krankenkassen übernehmen die Kosten der Behandlung, wenn der Schweregrad in mindestens einer Ursachengruppe den Grad 3 erreicht (Abb. 1). Eine einzelne Abweichung Grad 3 kann jedoch eine geringere (zahn-)medizinische Bedeutung haben als mehrere gleichzeitig auftretende Abweichungen geringeren Schweregrades.

(2) Es findet keine Berücksichtigung ästhetischer, psychosozial relevanter Faktoren statt, wie beispielsweise bei der ästhetischen Komponente des IOTN-Index.

(3) Es gibt keine Ausnahmeregelung für kieferorthopädische Leistungen, wie z. B. für die Entfernung von Weisheitszähnen. Eine Entfernung der Weisheitszähne in Intubationsnarkose ist eigentlich keine Regelleistung, bei Vorlage eines psychologischen Gutachtens ist jedoch eine Durchführung zulasten der GKV möglich.

## Inanspruchnahme kieferorthopädischer Leistungen

Trotz kleiner Schwächen ermöglichen die kieferorthopädischen Indikationsgruppen (KIG) auch im Vergleich zu anderen öffentlichen Gesundheitssystemen Mitteleuropas eine breitflächige Versorgung der Bevölkerung mit kieferorthopädischen Leistungen [12, 35]. Gemäß den Ergebnissen der Studie des Robert Koch-Instituts zur Gesundheit von Kindern und Jugendlichen in Deutschland (KiGGS Welle 2) sind 25,8 % aller Mädchen und 21,1 % aller Jungen im Alter von 3–17 Jahren in aktiver kieferorthopädischer Behandlung [36]. Im Vergleich zur KiGGS-Basiserhebung vor rund 10 Jahren ist die Häufigkeit der Inanspruchnahme unter den 7‑ bis-17-Jährigen um 9,1 % gestiegen, dies entspricht dem allgemeinen Trend zur höheren Inanspruchnahme zahnärztlicher (+5,8 %; [37]) sowie allgemeiner Vorsorgeuntersuchungen im Kindes- und Jugendalter (+15,6 %; [38]).

## Qualität der kieferorthopädischen Versorgung

Ein international anerkanntes evidenzbasiertes Bewertungsschema für die Beurteilung der Qualität kieferorthopädischer Behandlungen existiert bis dato nicht [39]. Ferner gibt es national wie international bisher nur vereinzelte Studien zur Qualität der kieferorthopädischen Versorgung, die leider in der Regel die epidemiologischen Voraussetzungen für eine Generalisierbarkeit auf das jeweilige Gesundheitssystem nicht erfüllen (Tab. 1). Eine häufig zur Beurteilung kieferorthopädischer Behandlungsergebnisse verwendete Methode ist der Peer-Assessment-Rating-Index (PAR-Index), bei dem der Schweregrad von Zahn- und Kieferfehlstellungen durch einen Summenscore, also eine einzige Zahl, ausgedrückt wird. Je höher der PAR-Score, desto schwerwiegender ist die Zahn- und Kieferfehlstellung. Ein hoher Qualitätsstandard kieferorthopädischer Versorgung in öffentlichen Gesundheitssystemen ist gekennzeichnet durch eine mittlere prozentuale Reduktion des gewichteten PAR-Index von > 70 % und einen geringen Prozentsatz von Fällen, in denen keine Verbesserung oder sogar eine Verschlechterung eintrat (Worse‑/No-different-Fälle; [49, 50]). Die bisher für Deutschland vorliegenden Studien erfüllen diese Anforderungen ganz oder teilweise und müssen den europäischen Vergleich nicht scheuen (Tab. 1), ihre Ergebnisse sind jedoch nicht generalisierbar.

**Table 1:** 

Publikationsjahr	Autoren	Untersuchte Region	Ziehung Stichprobe/Anzahl Praxen^a^	Anzahl Patienten	Mittlere PAR-Score-Reduktion (%)^b^	Anteil Fälle (%) „worse/no different“
1990	Richmond [40]	England und Wales	Systematisch (5 %)	1010	55,0	20
1993	Richmond & Andrews [41]	Norwegen	6	220	78,0	\
1997	Birkeland et al. [42]	Norwegen	1	224	76,7	3,7
1999	Turbill et al. [43]	England und Wales	Systematisch (2 %)	1527	47,6	\
2000	Teh et al. [44]	Schottland	Randomisiert	128	59,2	14,8
2009	Deans et al. [45]	Europa	10 Praxen in 7 Ländern	429	\	9,5
2017	Klaus et al. [46]	*Deutschland*	1	1653	\	3,4
2017	von Bremen et al. [35]	*Deutschland*	1	5385	\	7,6
2018	Isherwood et al. [47]	Schottland	38	1140	87,7	0,0
2021	Graf et al. [48]	*Deutschland*	7	335	83,5	0,9

^a^Informationen zur Ziehung der Stichprobe (% einer vordefinierten Grundgesamtheit) bzw. der Anzahl der Praxen aus denen die Patienten rekrutiert wurden

^b^PAR-Score: Peer-Assessment-Rating. Methode zur Beurteilung kieferorthopädischer Behandlungsergebnisse, wobei der Schweregrad von Zahn- und Kieferfehlstellungen in einen Summenscore angegeben wird

Bezüglich der Kosteneffektivität gibt es nur eine einzige explorative Studie mit paneuropäischer Perspektive [45]. Bezüglich der kaufkraftadjustierten Kosteneffektivität (ICON-Punktreduktion pro €) lagen die deutschen Praxen im mittleren Bereich der 7 untersuchten europäischen Länder.

## Fazit und Ausblick

Zahn- und Kieferfehlstellungen haben eine hohe gesundheitliche Relevanz. Die kieferorthopädische Behandlung dieser Fehlstellungen ist ein unverzichtbarer und integraler Bestandteil einer umfassenden zahnmedizinischen und medizinischen Versorgung der Bevölkerung.

Zur Verbesserung des bestehenden Versorgungsniveaus von Zahn- und Kieferfehlstellungen wäre die Aufnahme eines kieferorthopädischen Screenings im 7.–8. Lebensjahr in den durch den gemeinsamen Bundesausschuss (G-BA) festgelegten systematischen, zahnärztlichen Früherkennungskatalog wünschenswert. Diese Untersuchung sollte idealerweise durch Fachzahnärzte für Kieferorthopädie erfolgen.

## References

[1] Sheiham A (2005). Oral health, general health and quality of life. Bull World Health Organ.

[2] Dörfer C, Benz C, Aida J, Campard G (2017). The relationship of oral health with general health and NCDs: a brief review. Int Dent J.

[3] Dhar V, Jain A, Van Dyke TE, Kohli A (2007). Prevalence of gingival diseases, malocclusion and fluorosis in school-going students of rural areas in Udaipur district. J Indian Soc Pedod Prev Dent.

[4] Alhammadi MS, Halboud E, Fayed MS, Labib A, El-Saaidi C (2018). Global distribution of malocclusion traits: a systematic review. Dental Press J Orthod.

[5] Bundeszahnärztekammer (2016) Muster-Weiterbildungsordnung der Bundeszahnärztekammer. https://www.bzaek.de/fileadmin/PDFs/b/mwbo.pdf/. Zugegriffen: 10. Juni 2020

[6] Huh A, Horton MJ, Cuenco KT, Raoul G, Rowlerson AM, Ferri J, Sciote JJ (2013). Epigenetic influence of KAT6B and HDAC4 in the development of skeletal malocclusion. Am J Orthod Dentofacial Orthop.

[7] Hartsfield JK, Jacob GJ, Morford LA (2017). Heredity, genetics and orthodontics—how much has this research really helped?. Semin Orthod.

[8] D’Onofrio LD (2018). Oral dysfunction as a cause of malocclusion. Orthod Craniofac Res.

[9] Institut der Deutschen Zahnärzte (1991). Mundgesundheitszustand und -verhalten in der Bundesrepublik Deutschland.

[10] Schopf P (2003). Indication for and frequency of early orthodontic therapy or interceptive measures. J Orofac Orthop.

[11] Tausche E, Luck O, Harzer W (2004). Prevalence of malocclusions in the early mixed dentition and orthodontic treatment need. Eur J Orthod.

[12] Glasl B, Ludwig B, Schopf P (2006). Prevalence and development of KIG-relevant symptoms in primary school children from Frankfurt am Main. J Orofac Orthop.

[13] Lux CJ, Dücker B, Pritsch M, Komposch G, Niekusch U (2009). Occlusal status and prevalence of occlusal malocclusion traits among 9-year-old schoolchildren. Eur J Orthod.

[14] Favero L, Arreghini A, Cocilovo F, Favero V (2013). Respiratory disorders in paediatric age: orthodontic diagnosis and treatment in dysmetabolic obese children and allergic slim children. Eur J Paediatr Dent.

[15] Souto-Souza D, Soares MAC, Primo-Miranda EF, Pereira LJ, Ramos-Jorge ML, Ramos-Jorge J (2020). The influence of malocclusion, sucking habits and dental caries in the masticatory function of preschool children. Braz oral res.

[16] Koskela A, Neittaanmäki A, Rönnberg K, Palotie A, Ripatti S, Palotie T (2020). The relation of severe malocclusion to patients’ mental and behavioral disorders, growth and speech problems. Eur J Orthod.

[17] Manfredini D, Lombardo L, Siciliani G (2017). Temporomandibular disorders and dental occlusion. A systematic review of association studies: end of an era?. J Oral Rehabil.

[18] Petti S (2015). Over two hundred million injuries to anterior teeth attributable to large overjet: a meta-analysis. Dent Traumatol.

[19] Bernhardt O, Krey KF, Daboul A, Völzke H, Kindler S, Kocher T, Schwahn Ch (2019). New insights in the link between malocclusion and periodontal disease. J Clin Periodontol.

[20] Ferrando-Magraner E, Garcia-Sanz V, Bellot-Arcis C, Montiel-Company JM, Almerich-Silla JM, Paredes-Gallardo V (2019). Oral health-related quality of life of adolescents after orthodontic treatment. A systematic review. J Clin Exp Dent.

[21] Kunz F, Platte P, Keß S, Geim L, Zeman F, Proff P, Hirschfelder U, Stellzig-Eisenhauer A (2019). Impact of specific orthodontic parameters on the oral health-related quality of life in children and adolescents. J Orofac Orthop.

[22] Tristao SCPC, Magno MB, Vaz Braga Pinto A, Christovam IFO, Ferreira DMTP, Cople Maia L, Ribeiro de Souza IP (2020). Is there a relationship between malocclusion and bullying? A systematic review. Prog Orthod.

[23] Kunz F, Platte P, Keß S, Geim L, Zeman F, Proff P, Hirschfelder U, Stellzig-Eisenhauer A (2018). Correlation between oral health-related quality of life and orthodontic treatment need in children and adolescents—a prospective interdisciplinary multicenter cohort study. J Orofac Orthop.

[24] Nguee AAM, Ongkosuwito EM, Jaddoe VWV, Wolvius EB, Kragt L (2020). Impact of orthodontic treatment need and deviant occlusal traits on oral health-related quality of life in children: a cross-sectional study in the generation R cohort. Am J Orthod Dentofacial Orthop.

[25] Macey R, Thiruvenkatachari B, O’Brien K, Batista KBSL (2020). Do malocclusion and orthodontic treatment impact oral health? A systematic review and meta-analysis. Am J Orthod Dentofacial Orthop.

[26] Ruf S (2017). Standard ohne Gold. RCT Studien scheitern oft an klinischen Fragestellungen der Kieferorthopädie. Zahnmed Ges.

[27] DGKFO (2018) Positionspapier der Deutschen Gesellschaft für Kieferorthopädie zum Medizinischen Nutzen kieferorthopädischer Behandlungen. https://www.dgkfo-vorstand.de/fileadmin/redaktion/upload_vorstand/Praesiordner/Bundesrechnungshof/DGKFO-Positionspapier_Nutzen_der_KFO.pdf. Zugegriffen: 06-04-2021

[28] Lima Araujo BC, de Magalhaes Simoes S, de Gois-Santos VT, Saquete Martins-Filho PR (2020). Association between mouth breathing and asthma: a systematic review and meta-analysis. Curr Allergy Asthma Rep.

[29] Suzuki J, Shimazaki K, Koike S, Ono T (2018). Gastric emptying rate before and after orthodontic treatment examined with the [^13^C] breath test: a pilot study. Am J Orthod Dentofacial Orthop.

[30] Doshi UH, Bhad-Patil WA (2011). Speech defect and orthodontics: a contemporary review. Orthodontics (Chic.).

[31] Brook PH, Shaw WC (1989). The development of an index of orthodontic treatment priority. Eur J Orthod.

[32] Daniels C, Richmond S (2000). The development of the index of complexity, outcome and need (ICON). J Orthod.

[33] Linder-Aronson S (1974) Orthodontics in the Swedish public dental health service. Trans Eur Orthod Soc :233–240 4534974

[34] Bundesamt für Sozialversicherung (BSV), Schweizerische Zahnärzte-Gesellschaft (SSO) (2009) Informationen für Zahnärzt und Zahnärzte über die Eidgenössische Invaliditätsversicherung. https://www.sso.ch/fileadmin/upload_sso/2_Zahnaerzte/1_Informationen/Zaz-Infos_BSV_IV_SSO_Nov_2010_D_2_.pdf. Zugegriffen: 10. Juni 2020

[35] von Bremen J, Streckbein EM, Ruf S (2017). Changes in university orthodontic care over a period of 20 years. J Orofac Orthop.

[36] Seeling S, Prütz F (2018). Inanspruchnahme kieferorthopädischer Behandlung durch Kinder und Jugendliche in Deutschland – Querschnittsergebnisse aus KiGGS Welle 2 und Trends. J Health Monit.

[37] Krause L, Kuntz B, Schenk L, Knopf H (2018). Mundgesundheitsverhalten von Kindern und Jugendlichen in Deutschland – Querschnittsergebnisse aus KiGGs Welle 2 und Trends. J Health Monit.

[38] Schmidtke C, Kuntz B, Starker A, Lampert T (2018). Inanspruchnahme der Früherkennungsuntersuchungen für Kinder in Deutschland – Querschnittsergebnisse aus KiGGs Welle 2. J Health Monit.

[39] Borzabadi-Farahani A (2011) An overview of selected orthodontic treatment need indices. Principles in contemporary orthodontics. http://www.intechopen.com/books/principles-in-contemporary-orthodontics/an-overview-of-selected-orthodontic-treatment-need-indices. Zugegriffen: 06-04-2021

[40] Richmond S (1990). A critical evaluation of orthodontic treatment in general dental services of England and Wales.

[41] Richmond S, Andrews M (1993). Orthodontic treatment standards in Norway. Eur. J. Orthod..

[42] Birkeland K, Furevik J, Boe OE, Wisth PJ (1997). Evaluation of treatment and post-treatment changes by the PAR Index. Eur J Orthod.

[43] Turbill EA, Richmon S, Wright JL (1999). A closer look at general dental service orthodontics in England and Wales. I: factors influencing effectiveness. Br Dent J.

[44] Teh LH, Kerr WJS, McColl JH (2000). Orthodontic treatment with fixed appliances in the general dental service in Scotland. J Orthod.

[45] Deans J, Playle R, Durning P, Richmond S (2009). An explorative study of cost-effectiveness of orthodontic care in seven European countries. Eur J Orthod.

[46] Klaus K, Stark P, Serbesis TSP, Pancherz H, Ruf S (2017). Excellent versus unacceptable orthodontic results: influencing factors. Eur J Orthod.

[47] Isherwood G, Pencovich R, Burnside G, Miller S (2018). The Scottish orthodontic peer review project: the outcome of treatment and standard of record keeping by orthodontic specialist practitioners in Scotland. J Orthod.

[48] Graf I, Bock NC, Bartzela T, Röper V, Schumann U, Reck K, Christ H, Höfer K, Fritz U, Wiechmann D, Jost-Brinkmann PG, Wolf M, Ruf S, Braumann B (2021) Quality of orthodontic care—a multicenter cohort study in Germany. Part 1: evaluation of effectiveness of orthodontic treatments and predictive factors. J Orofac Orthop. 10.1007/s00056-021-00304-3 10.1007/s00056-021-00304-3 PMC9395451 34142175

[49] Richmond S, Shaw WC, O’Brien KD, Buchanan IB, Jones R, Stephens CD, Roberts CT, Andrews M (1992). The development of the PAR index (peer assessment rating): reliability and validity. Eur J Orthod.

[50] Richmond S, Shaw WC, Roberts CT, Andrews M (1992). The PAR index (peer assessment rating): methods to determine outcome of orthodontic treatment in terms of improvement and standards. Eur J Orthod.

